# Volatile Metabolomics Reveals the Effects of Different Bagging Types on Peel Aroma of ‘Siam Red Pomelo’

**DOI:** 10.3390/foods15203608

**Published:** 2026-10-10

**Authors:** Qinlin Deng, Xiaojiao Zhang, Caihong Zhang, Xue Li, Xiangzeng Xu, Shide Gao

**Affiliations:** Yunnan Institute of Tropical Crops, Jinghong 666100, China

**Keywords:** *Citrus maxima*, fruit bagging, HS-SPME-GC-MS, volatile organic compounds, rOAV, flavoromics

## Abstract

Fruit bagging is a common horticultural practice, but its effects on citrus peel volatile metabolism remain poorly understood. *Citrus maxima* ‘Siam Red Pomelo’ is a distinctive red-skinned pomelo germplasm, and the influence of different bagging types on its peel volatile composition has not been systematically characterized. In this study, peel volatiles from fruit subjected to a non-bagged control (CK), a white nonwoven bag (W), and a yellow–black double-layer paper bag (B) were profiled using HS-SPME–GC–MS. Relative odor activity values (rOAVs), calculated from single-internal-standard semi-quantitative VOC estimates and literature-reported odor thresholds, were used to prioritize compounds with potential aroma relevance. A total of 1598 volatile organic compounds were identified, mainly terpenoids and esters. The two bagging treatments produced distinct volatile metabolic profiles. The B treatment showed relatively greater contributions of several sesquiterpenoids and oxygenated derivatives, including 7-epi-α-eudesmol and caryophyllene oxide, together with reduced contributions of several green-associated monoterpenes. In contrast, the W treatment showed relatively greater contributions of monoterpene esters, such as linalyl butyrate, associated with database-reported floral and fruity odor descriptors. KEGG enrichment indicated treatment-associated changes in terpenoid biosynthesis and butanoate metabolism. Overall, different bag types were associated with distinct peel volatile profiles and distributions of putative aroma-related compounds. Because the rOAVs were based on semi-quantitative data and no GC–olfactometry, aroma recombination/omission experiments, or sensory evaluation was performed, the inferred aroma-related differences should be regarded as hypotheses requiring sensory validation.

## 1. Introduction

Citrus is one of the most important fruit crops worldwide and is highly valued by consumers for its distinctive aroma, rich nutritional profile, and well-recognized health benefits [1]. China is the world’s largest citrus-producing country, with a total production of 67.92 million tons in 2024, accounting for more than one-third of global citrus production. In addition, international trade in major citrus products generated a trade surplus of approximately USD 1.0 billion (FAO database and National Bureau of Statistics of China, 2025). Citrus germplasm resources are fundamental to genetic improvement, the dissection of quality traits, and the mining of elite genes [2]. To the best of our knowledge, the red-skinned ‘Siam Red Pomelo’ (*Citrus maxima*) is a pomelo germplasm that has not been systematically characterized and is morphologically distinct from the previously described green-skinned, red-fleshed cultivar ‘Tubtim Siam’ (Siam Ruby) [3]. Field observations revealed that the peel of this accession possesses a distinctive and relatively intense aroma, suggesting its potential as a natural source of aroma compounds. However, systematic studies on this germplasm remain lacking, and its peel volatile profile and the VOCs potentially relevant to its characteristic aroma have not yet been systematically prioritized or sensorially validated.

Fruit aroma is a key determinant of citrus sensory quality and is primarily governed by the accumulation of volatile compounds. Plant volatiles are generally low-molecular-weight metabolites with sufficient vapor pressure to enter the headspace under normal environmental conditions [4]. They not only impart characteristic aromas and flavors to fruits, thereby shaping consumer perception and purchasing behavior, but also exhibit diverse biological activities. In particular, volatile compounds function as important chemical signals in interactions between plants and neighboring plants, microorganisms, insects, and the surrounding environment [5]. In citrus fruits, volatile compounds are predominantly stored in the oil glands of the flavedo, with terpenoids representing the largest fraction in both abundance and diversity. D-Limonene, valencene, and linalool are among the most representative volatile constituents [6]. Nevertheless, substantial variation in bioactive constituents exists among citrus cultivars, reflecting the extensive diversity of citrus germplasm resources [1].

The volatile composition of citrus fruit is strongly influenced by genotype, tissue type, developmental stage, and tree age, resulting in considerable variation in aroma profiles among citrus germplasm. Despite this diversity, previous studies consistently indicate that terpenoids, particularly monoterpenes, constitute a major component of citrus volatiles [7]. However, the compounds associated with characteristic aroma differ substantially among cultivars. For example, D-limonene predominates in *Citrus aurantium* Changshan-huyou and Jinggang honey pomelo, whereas comparative analyses of lemon and *Citrus medica* cultivars have revealed distinct combinations of alcohols, aldehydes, esters, terpenoids, and other potent odorants associated with cultivar-specific aroma characteristics [8,9,10]. Developmental and physiological factors further modify these profiles. Monoterpene accumulation generally increases during fruit maturation in navel orange, whereas tree age in *Citrus reticulata* cv. Chachiensis affects different volatile classes and individual sesquiterpenes in distinct directions [11,12,13]. Collectively, these findings indicate that citrus aroma cannot be represented by a universal volatile profile but instead reflects genotype-specific and development-dependent regulation. Nevertheless, previous studies have focused predominantly on widely cultivated citrus types such as lemon, mandarin, and sweet orange, whereas distinctive germplasm possessing unusual fragrance characteristics remains comparatively underexplored.

Fruit bagging is a widely adopted orchard management practice in the production of citrus, pear, peach, grape, apple, and other fruit crops [14,15,16,17,18]. By establishing a relatively enclosed physical barrier around the developing fruit, bagging can improve peel appearance, reduce mechanical injury and pest or disease damage, and minimize pesticide residues [14]. However, its effects on aroma are not consistent across crops or bagging treatments. In peach, bagging reduced C6 compounds, aldehydes, esters, and total volatiles, whereas studies in grape showed both decreases and increases in specific volatile compounds [18,19]. Moreover, different bag colors or materials produced contrasting effects on volatile classes: some treatments promoted ester accumulation while suppressing aldehydes, alcohols, terpenes, and ketones, whereas other studies reported a greater relative contribution of aldehydes in bagged fruit [15,20]. In Meyer lemon, bagging did not substantially alter the overall chemical-class composition but shifted the timing of maximum volatile accumulation [16]. These contrasting responses demonstrate that the effects of bagging on fruit aroma are highly context-dependent and cannot be generalized across species or bagging materials. Importantly, evidence from citrus remains limited, and the extent to which differences in bagging material and associated light transmittance regulate volatile metabolism and aroma quality has not been systematically resolved.

HS-SPME–GC–MS has become a widely used approach for profiling volatile organic compounds in citrus and other horticultural products because of its high sensitivity, minimal sample preparation, and suitability for broad volatile screening [21,22,23,24]. Recent applications have revealed extensive volatile diversity among citrus cultivars and production environments [8,25,26]. However, compound abundance alone does not necessarily reflect sensory importance because volatile compounds occurring at relatively low concentrations may contribute strongly to aroma when their odor thresholds are low. Integrating relative odor activity values (rOAVs) with HS-SPME–GC–MS profiling therefore provides a more informative means of distinguishing abundant metabolites from compounds that may potentially contribute to aroma [26,27]. To date, such integrated flavoromic approaches have been applied mainly to mandarins, sweet oranges, and related citrus groups, whereas systematic characterization of aroma-active compounds in distinctive pomelo germplasm remains scarce.

Despite these advances, how different fruit bag types affect volatile metabolite composition and aroma-related characteristics in specialty citrus remains poorly understood. Therefore, the present study aimed to (i) comprehensively profile the volatile organic compounds in the peel of ‘Siam Red Pomelo’ and screen candidate aroma-active constituents using HS-SPME–GC–MS combined with relative odor activity value analysis; (ii) compare the effects of a non-bagged control, a white nonwoven fabric bag treatment, and a yellow–black double-layer paper bag treatment on volatile metabolite composition and aroma-related volatile characteristics; and (iii) explore the metabolic pathways associated with bagging-treatment-specific differences in volatile composition. The findings are expected to provide a scientific basis for understanding how different bagging practices are associated with variation in peel volatile traits.

## 2. Materials and Methods

### 2.1. Plant Materials and Experimental Treatments

The experimental material consisted of fruits of the ‘Siam Red Pomelo’ (total soluble solids (TSS) > 7% and titratable acidity (TA) < 0.3%), harvested from the Yunnan Institute of Tropical Crops, China (22°01′ N, 100°78′ E; elevation: 540 m), where the orchard is equipped with an integrated fertigation system. Three fruit-bagging treatments were established: a yellow–black double-layer paper bag treatment (B), a white nonwoven fabric bag treatment (W), and a non-bagged control (CK).

Fifteen healthy trees exhibiting uniform growth vigor were selected for each treatment. On each tree, fifteen fruits with uniform size and regular shape were randomly selected, individually labeled, and subjected to bagging treatment. At commercial maturity, six trees were randomly selected from the fifteen trees of each treatment for volatile metabolomics analysis. Peel tissues were collected from multiple fruits of each selected tree, pooled as one sample, immediately flash-frozen in liquid nitrogen, and stored at −80 °C until subsequent volatile metabolomics analysis. Thus, six independent biological replicates were obtained for each treatment. The peel tissues were excised from the equatorial region of the fruit, with a thickness of approximately 2–3 mm and an area of approximately 1 × 1 cm^2^.

### 2.2. Metabolites Extraction

The volatile metabolite extraction procedure used in the present study was performed with reference to the previously described method [28]. After cryogenic processing, 500 mg of each sample was transferred into a 20 mL headspace vial (Agilent, Palo Alto, CA, USA) containing 2 mL of saturated sodium chloride solution, which was added to inhibit enzymatic reactions. The vials were then immediately sealed using crimp caps equipped with TFE–silicone septa (Agilent). Prior to SPME extraction, the sealed vials were maintained at 60 °C for 5 min to allow thermal equilibration. Subsequently, a 120 µm DVB/CWR/PDMS SPME Arrow fiber (Agilent) was inserted into the vial headspace and exposed for 15 min under the same temperature conditions. Each treatment included six biological replicates, and samples derived from all bagging treatments were subjected to an identical analytical procedure.

Following SPME extraction, volatile compounds retained on the SPME Arrow fiber coating were thermally released in the GC inlet of an Agilent 8890 system at 250 °C for 5 min. Subsequent chromatographic separation and mass spectrometric detection were conducted using an Agilent 8890 gas chromatograph coupled with a 7000D mass spectrometer (Agilent Technologies, Inc., Santa Clara, CA, USA). The analytical system was equipped with a DB-5MS fused-silica capillary column (30 m × 0.25 mm i.d. × 0.25 μm film thickness) coated with a 5% phenyl-polymethylsiloxane stationary phase. Helium was employed as the carrier gas under a constant flow rate of 1.2 mL/min, while the injector temperature was maintained at 250 °C.

### 2.3. GC–MS Conditions and Qualitative and Semi-Quantitative Analysis of Metabolites

The GC oven was programmed with an initial temperature of 40 °C, held for 3.5 min, followed by a temperature increase to 100 °C at a rate of 10 °C/min, then raised to 180 °C at 7 °C/min, and subsequently elevated to 280 °C at 25 °C/min, with the final temperature maintained for 5 min. Mass spectrometric data were collected using electron ionization (EI) at 70 eV. The temperatures of the quadrupole analyzer, ion source, and transfer line were maintained at 150, 230, and 280 °C, respectively. Qualitative identification and semi-quantitative determination of volatile compounds were performed using the selected ion monitoring (SIM) acquisition mode, in which specific characteristic ions of target compounds were selectively monitored to improve detection sensitivity and analytical specificity. Volatile compounds were annotated by matching their mass spectra against the MWGC and NIST databases and by comparing their linear retention indices (LRIs) with reference values provided by Metware Biotechnology (Wuhan, China) [29,30].

For semi-quantitative analysis, a known amount of the isotope-labeled internal standard 3-hexanone-2,2,4,4-d_4_ was added to each sample prior to HS-SPME–GC–MS analysis. The semi-quantitative content of each volatile compound was calculated based on the ratio of its chromatographic peak area to that of the internal standard, as described in previous studies [31], as follows:

X_i_ = (V_s_ × C_s_/M) × (I_i_/I_s_) × 10^−3^

where X_i_ is the semi-quantitative content of volatile compound i (μg/g); V_s_ is the volume of the internal standard solution added (μL); C_s_ is the concentration of the internal standard solution (μg/mL); M is the sample mass (g); I_i_ is the chromatographic peak area of volatile compound i; and I_s_ is the chromatographic peak area of the internal standard. Because a single isotope-labeled internal standard rather than compound-specific calibration curves and analyte-specific response factors was used, the X_i_ values represent internal-standard-based semi-quantitative estimates rather than absolute analyte concentrations. The factor 10^−3^ is solely a unit-conversion factor: V_s_ is recorded in μL whereas C_s_ is expressed in μg/mL, and 1 μL = 10^−3^ mL. Therefore, (V_s_ × 10^−3^) × C_s_ gives the mass of internal standard added in μg; dividing by M (g) and multiplying by the dimensionless peak-area ratio I_i_/I_s_ yields X_i_ in μg/g FW. The 10^−3^ term is not an empirical correction or normalization factor. Quality control (QC) samples, prepared by pooling equal volumes of all samples and analyzed at regular intervals, were included to monitor analytical stability.

### 2.4. Differential Metabolites Analysis

Differential metabolites in pairwise comparisons were screened according to two criteria: a variable importance in projection (VIP) value exceeding 1 and an absolute log2 fold change (|log2FC|) ≥ 1.0. For comparisons involving multiple experimental groups, candidate discriminatory metabolites were selected based on a VIP score > 1 together with statistical significance determined by one-way ANOVA (*p* < 0.05).

The VIP values were obtained from an orthogonal partial least squares discriminant analysis (OPLS-DA) model constructed using the R package MetaboAnalystR (v4.2.0), which was also employed to generate model score plots and permutation validation results. Prior to OPLS-DA analysis, the metabolite dataset was subjected to log2 transformation followed by unit variance scaling. The robustness of the model was evaluated through a permutation test involving 200 randomized iterations to minimize the possibility of model overfitting.

### 2.5. KEGG Annotation and Pathway Enrichment Analysis

Metabolite identification was performed through database-based annotation against the KEGG Compound repository (http://www.kegg.jp/kegg/compound/ (accessed on 1 June 2026)). The annotated metabolites were then assigned to corresponding biological pathways by conducting pathway mapping using the KEGG Pathway database (http://www.kegg.jp/kegg/pathway.html (accessed on 1 June 2026)).

### 2.6. Relative Odor Activity Value (rOAV) Analysis

The relative odor activity value (rOAV) represents an integrated parameter that combines the measured concentration of volatile compounds with their corresponding odor thresholds, thereby providing an estimate of their potential contribution to the overall aroma profile. This approach has been extensively applied for the screening and identification of aroma-active constituents in food matrices.

In this study, rOAV values were determined following previously reported procedures using the equation: rOAV_i_ = C_i_/T_i_, where C_i_ represents the semi-quantitative concentration of volatile feature i (µg/g for the solid pulp samples), and T_i_ represents the odor threshold value of the corresponding compound obtained from published literature [32]. The semi-quantitative concentrations were obtained using the internal-standard method. No additional normalization to the maximum odor-activity value within each treatment was performed.

The reported odor descriptors of the identified volatile organic compounds (VOCs) were annotated by consulting multiple specialized flavor and odor databases, including The Good Scents Company (http://www.thegoodscentscompany.com (accessed on 20 June 2026)), Odour.org.uk (http://www.odour.org.uk/odour/index.html (accessed on 20 June 2026)), Perflavory (http://perflavory.com/ (accessed on 20 June 2026)), and the Food Flavor Laboratory database (http://foodflavorlab.cn/ (accessed on 20 June 2026)) [28]. Only database- and literature-derived annotations were used for odor-descriptor assignment; sensory-panel data were not included in this analysis. Therefore, these descriptors represent database-derived annotations rather than experimentally verified sensory perceptions of the samples. For aroma-attribute analysis, differential VOCs identified in each pairwise comparison were mapped to all reported odor descriptors. Descriptor assignment was non-exclusive; when a compound was associated with multiple odor descriptors, all corresponding descriptors were retained. Aroma descriptors were ranked according to the number of associated differential VOCs, and the 10 most frequently represented descriptors were used for visualization.

Volatile compounds with rOAV ≥ 1 were retained as candidate aroma-contributing compounds for subsequent analysis. This threshold indicates only that the semi-quantitative estimate equals or exceeds the literature-reported odor threshold on the harmonized nominal concentration basis; it does not establish that the compound directly contributes to the perceived aroma. Because GC-olfactometry, aroma recombination/omission tests, and sensory-panel assessments were not conducted, all rOAV-based aroma interpretations remain hypotheses requiring sensory validation.

### 2.7. Statistical Analysis

All statistical evaluations were performed using one-way analysis of variance (ANOVA) implemented in GraphPad Prism 9. Experimental results are presented as mean ± standard deviation (SD). Differences were considered statistically significant at *p* < 0.05, while *p* values below 0.01 were interpreted as highly significant. Visualization of all experimental data was conducted using Metware Cloud, a freely available online platform for biological data analysis (https://cloud.metware.cn (accessed on 1 June 2026)).

## 3. Results

### 3.1. Effects of Different Bagging Treatments on Volatile Metabolites

**Figure 1:**
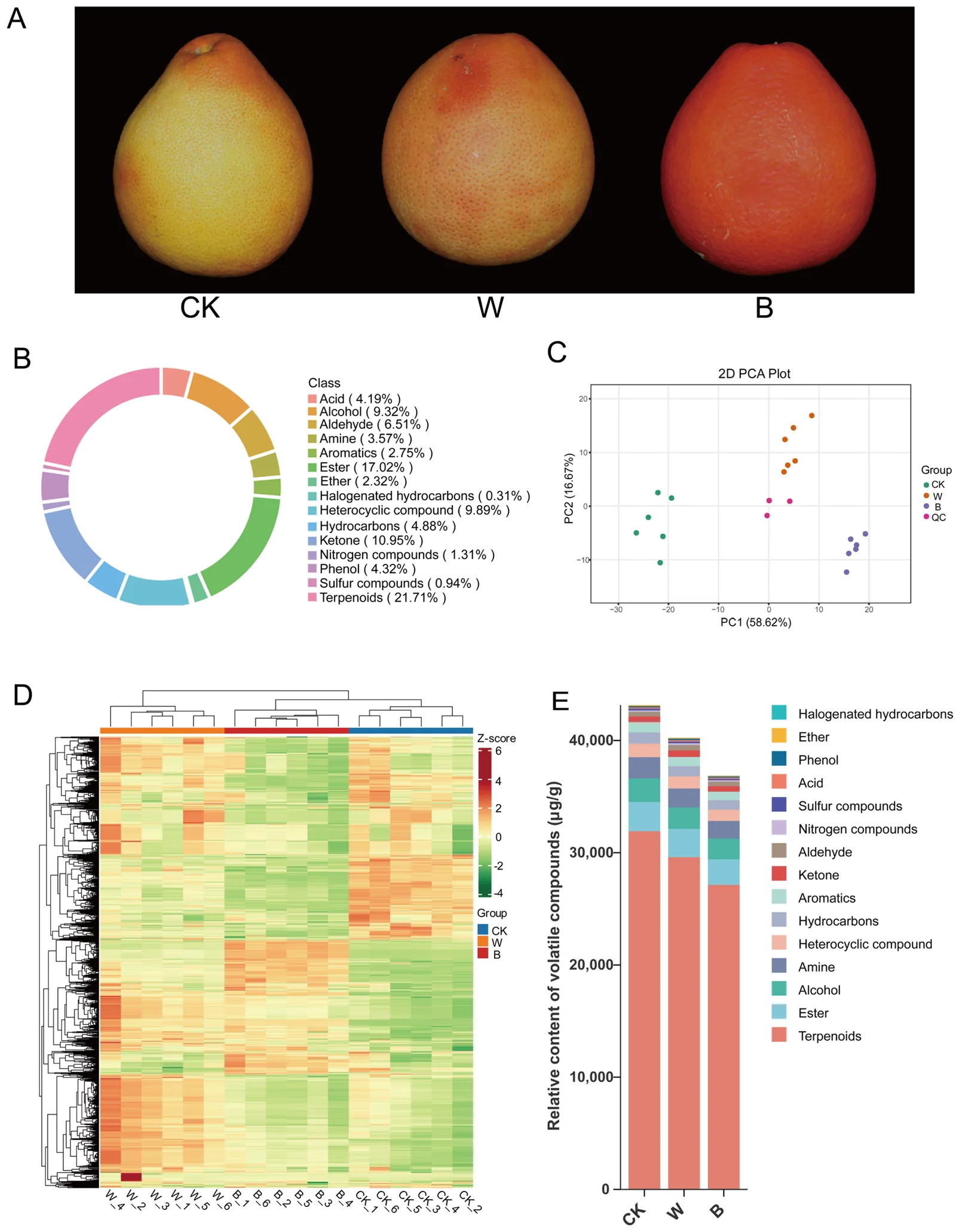
Volatile metabolite profiling of fruit under different bagging treatments. (**A**) Phenotypic characteristics of ‘Siam Red Pomelo’ under different bagging treatments. CK, non-bagged control; W, a white nonwoven fabric bag; B, a yellow–black double-layer paper bag. (**B**) Classification and composition of the identified metabolites. (**C**) Principal component analysis (PCA) score plot of samples based on the identified metabolites. QC, Quality control. (**D**) Hierarchical cluster analysis of volatile metabolites in the peel of Siam Red Pomelo subjected to different bagging treatments. (**E**) Summed semi-quantitative contents of different volatile organic compound (VOC) classes under different bagging treatments.

Volatile compounds constitute the chemical basis of fruit aroma. To investigate the effects of bagging on fruit aroma, volatile organic compounds in the fruit peel under the non-bagged control, white nonwoven fabric bag, and double-layer paper bag with a yellow exterior and black interior treatments were detected and analyzed using HS-SPME-GC-MS (Figure 1A). A total of 1598 volatile compounds belonging to 15 classes were identified, including 347 terpenoids, 272 esters, 175 ketones, 158 heterocyclic compounds, 149 alcohols, 104 aldehydes, 78 hydrocarbons, 69 phenols, 67 acids, 57 amines, 44 aromatic compounds, 37 ethers, 21 nitrogen-containing compounds, 15 sulfur-containing compounds, and 5 halogenated hydrocarbons (Figure 1B). Among all identified metabolites, terpenoids accounted for 21.71%, followed by esters (17.02%) and heterocyclic compounds (9.89%). Principal component analysis (PCA) was performed to investigate the overall metabolic differences among the groups and the variation among samples within each group. The results showed that the first principal component (PC1) and the second principal component (PC2) explained 58.62% and 16.67% of the total variance, respectively, and clearly distinguished the samples from the three bagging treatments (Figure 1C). Hierarchical cluster analysis further confirmed the PCA results (Figure 1D). Quality control (QC) samples, prepared by pooling equal volumes of all samples and analyzed at regular intervals, were included to monitor analytical stability. The QC samples clustered tightly, indicating good analytical reproducibility (Figure 1D). As shown in Figure 1E, the VOC profiles differed significantly among the three bagging treatments, with VOCs predominantly enriched in the non-bagged treatment.

### 3.2. Identification of Key Differential Volatile Metabolites

**Figure 2:**
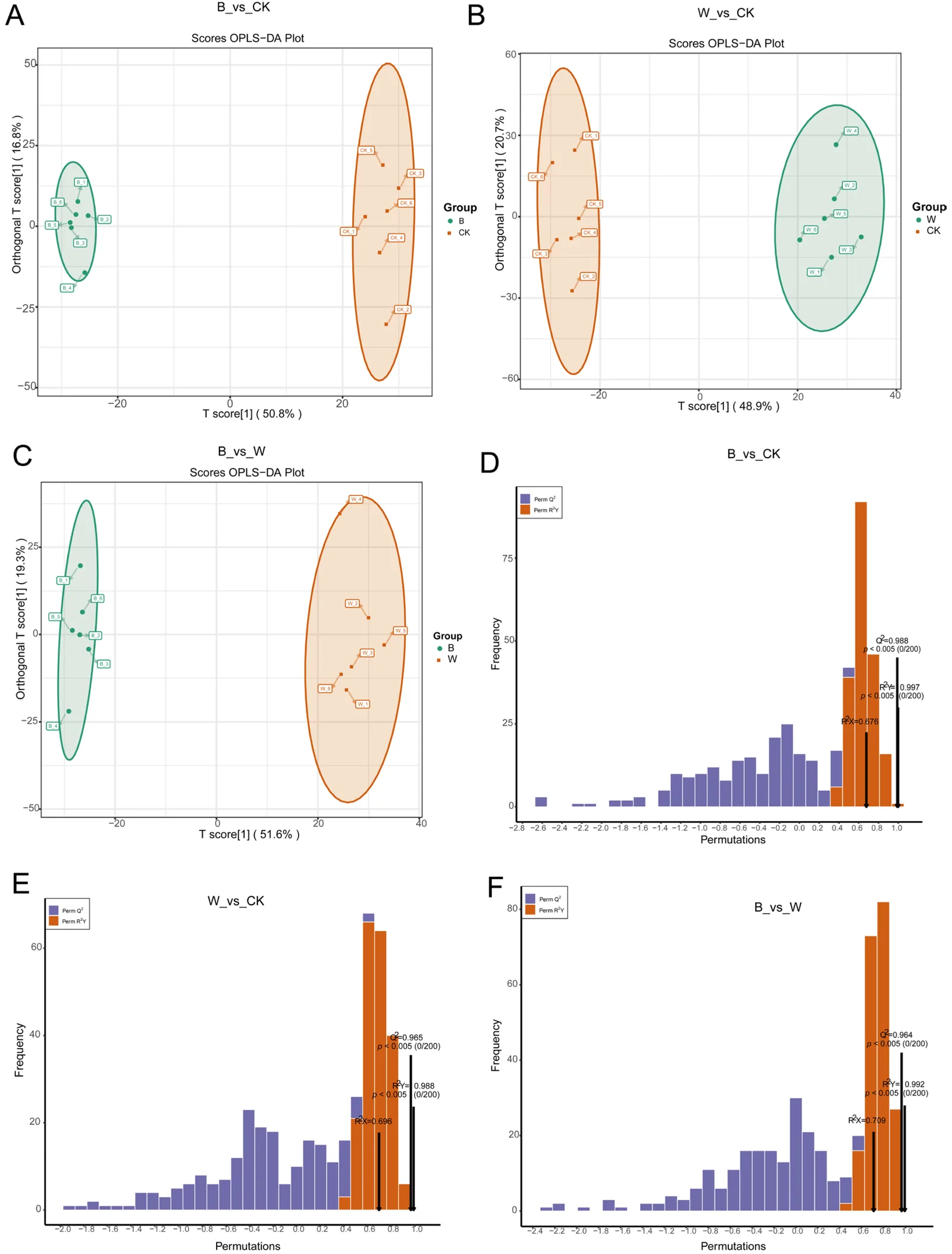
OPLS-DA of differential volatile metabolites in the peel of ‘Siam Red Pomelo’ under different bagging treatments. (**A**–**C**) Orthogonal partial least squares discriminant analysis (OPLS-DA) score plots of differential metabolites for the B_vs_CK, W_vs_CK, and B_vs_W comparisons, respectively. (**D**–**F**) Permutation test results for the OPLS-DA models corresponding to the B_vs_CK, W_vs_CK, and B_vs_W comparisons, respectively.

Although the major chemical-class composition remained broadly similar among treatments, the rOAV distributions of individual VOCs and their database-derived odor-descriptor associations differed among treatments of ‘Siam Red Pomelo’ peel. Clear separation was observed between B and CK and between W and CK, indicating that the volatile metabolic profiles of fruits subjected to either bagging treatment differed markedly from those of non-bagged fruits (Figure 2A–C). The OPLS-DA models were validated using permutation tests, which confirmed the absence of overfitting and demonstrated their good explanatory and predictive performance, thereby supporting further analysis (Figure 2D–F).

**Figure 3:**
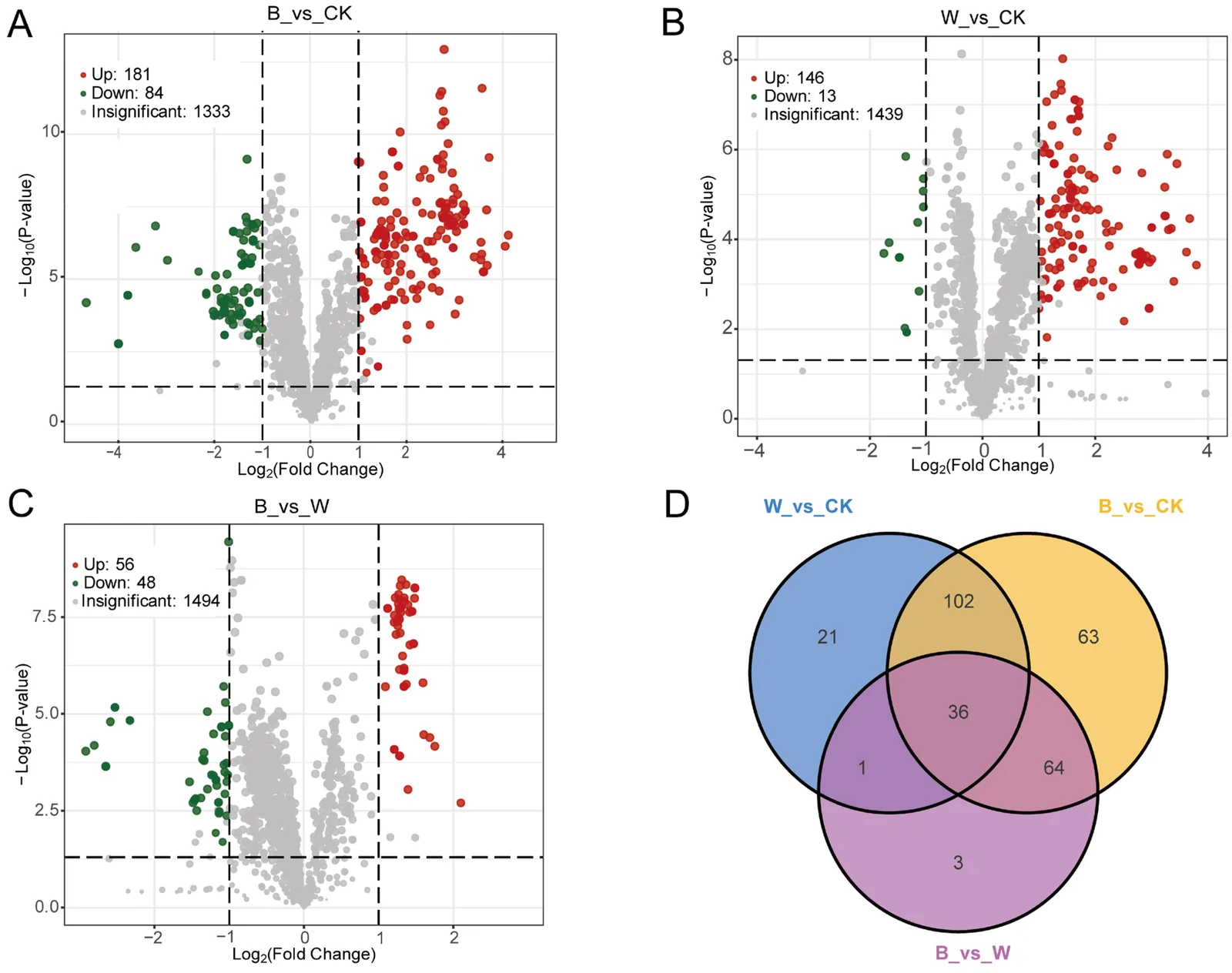
Screening of differential volatile metabolites (DVMs) in the peel of ‘Siam Red Pomelo’ under different bagging treatments. (**A**–**C**) Volcano plots of volatile metabolites for the B_vs_CK, W_vs_CK, and B_vs_W comparisons, respectively. (**D**) Venn diagram of differential volatile metabolites among the B_vs_CK, W_vs_CK, and B_vs_W comparisons.

Differential volatile metabolites (DVMs) were screened using different criteria according to the comparison strategy. For pairwise comparisons, DVMs were identified based on VIP > 1 and |log_2_(fold change)| ≥ 1, whereas for multiple-group comparisons, DVMs were identified based on VIP > 1 and one-way ANOVA (*p* < 0.05). Compared with CK, 181 DVMs were upregulated and 84 were downregulated in B (Figure 3A and Table S1). The upregulated DVMs mainly included terpenoids (33), alcohols (32), esters (25), and ketones (33) (Table S1), whereas the downregulated DVMs were primarily terpenoids (26) and esters (17). In the B vs. CK comparison, among the 20 top-ranked differential VOCs shown in Figure S1A, 14 exhibited increased accumulation, whereas six showed decreased accumulation. Notably, the 10 VOCs with the largest changes in accumulation included 2-acetyl-4,4-dimethylcyclopent-2-enone, benzimidazo[2,1-a]isoquinoline, isolongifolene, 4,5,9,10-dehydro-, santolina triene, bicyclo[2.2.1]hept-2-ene, 1,7,7-trimethyl-, ethanone, 1-(4-methylphenyl)-, ethanone, 1-(3-methylphenyl)-, 3H-indazol-3-one, 1-ethyl-1,2-dihydro-, 5-ethylhydantoin, and 2-pentyn-1-ol.

In W, 147 DVMs were upregulated and 13 were downregulated relative to CK (Figure 3B and Table S1), with the upregulated compounds consisting mainly of terpenoids (36) and esters (24). Compared with W, B contained 56 upregulated and 48 downregulated DVMs (Figure 3C and Table S1). The upregulated DVMs were dominated by alcohols, whereas the downregulated DVMs were mainly esters (Table S1). In the W vs. CK comparison, all 20 top-ranked differential VOCs displayed increased accumulation, as shown in Figure S1B. The 10 VOCs with the largest changes in accumulation included furan, 2,3-dihydro-, 3H-indazol-3-one, 1-ethyl-1,2-dihydro-, isolongifolene, 4,5,9,10-dehydro-, benzimidazo[2,1-a]isoquinoline, octanoic acid, methyl ester, piperonyl isobutyrate, 5-ethylhydantoin, 2-furancarboxylic acid, tetrahydro-3-methyl-5-oxo-, hydrazine, 1,1-bis(2-methylpropyl)-, and cis-3,5-diethyl-1,2,4-trithiolane. In the B vs. W comparison, among the 20 top-ranked differential VOCs shown in Figure S1C, nine exhibited increased accumulation, whereas 11 showed decreased accumulation. The 10 VOCs with the largest changes in accumulation included 2-acetyl-4,4-dimethylcyclopent-2-enone, 2-tetradecene, (E)-, santolina triene, bicyclo[2.2.1]hept-2-ene, 1,7,7-trimethyl-, n-butyl tiglate, 2,4-undecadienal, (E,E)-, 2,4-undecadienal, ethanone, 1-(4-methylphenyl)-, ethanone, 1-(3-methylphenyl)-, and benzaldehyde.

Venn diagram analysis showed that W vs. CK and B vs. CK shared the largest number of DVMs, with 102 metabolites. In addition, 63 DVMs were unique to B vs. CK, whereas 21 were unique to W vs. CK. A total of 36 DVMs were shared among all three pairwise comparisons, while only three were unique to B vs. W (Figure 3D and Table S1). These results indicate a substantial shared response to bagging relative to CK, together with additional treatment-specific differences between the W and B treatments.

### 3.3. K-Means Clustering Analysis

**Figure 4:**
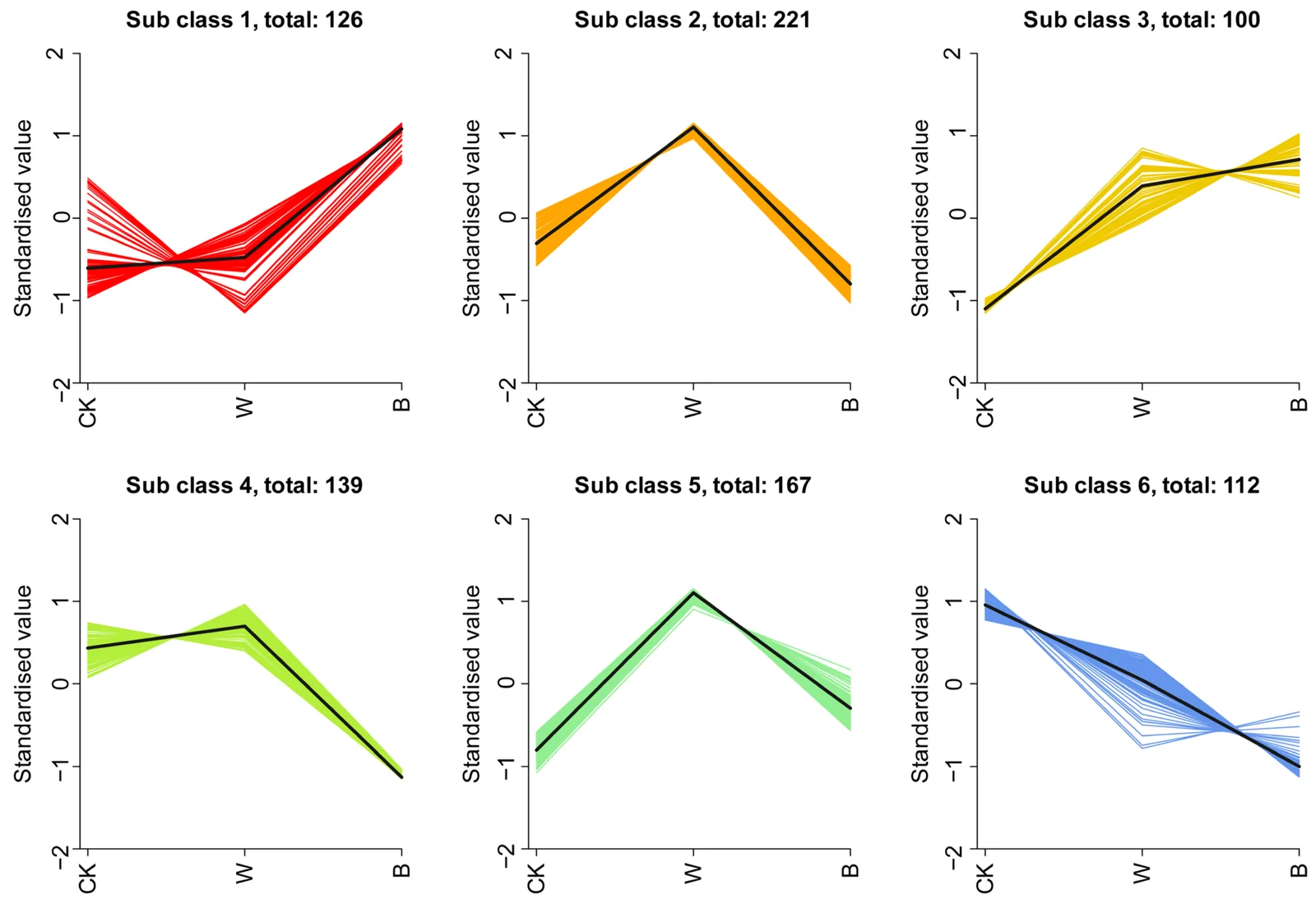
K-means clustering analysis of differential volatile metabolites in the peel of ‘Siam Red Pomelo’ under different bagging treatments. The vertical axis represents the standardized content of each metabolite, and the horizontal axis represents different samples. B: yellow–black double-layer paper bag treatment; W: white nonwoven fabric bag treatment; CK: non-bagged control.

Based on K-means clustering analysis, the differential volatile metabolites in the peel of ‘Siam Red Pomelo’ under the three bagging treatments were mainly classified into six distinct trends (Figure 4). Subclasses 2, 4, and 5 comprised metabolites whose abundances increased following the W treatment, whereas subclasses 1 and 3 contained metabolites that accumulated to higher levels under the B treatment than under the W treatment. Subclass 6 consisted of metabolites that exhibited the highest abundances in the CK and decreased in the bagging treatments. These results indicate that the W treatment mainly promoted the accumulation of metabolites in subclasses 2, 4, and 5, whereas the B treatment primarily enhanced the accumulation of metabolites in subclasses 1 and 3. Overall, the three bagging treatments produced distinct volatile metabolite accumulation patterns in pomelo peel.

### 3.4. KEGG Pathway Enrichment Analysis of Differentially Accumulated Metabolites

**Figure 5:**
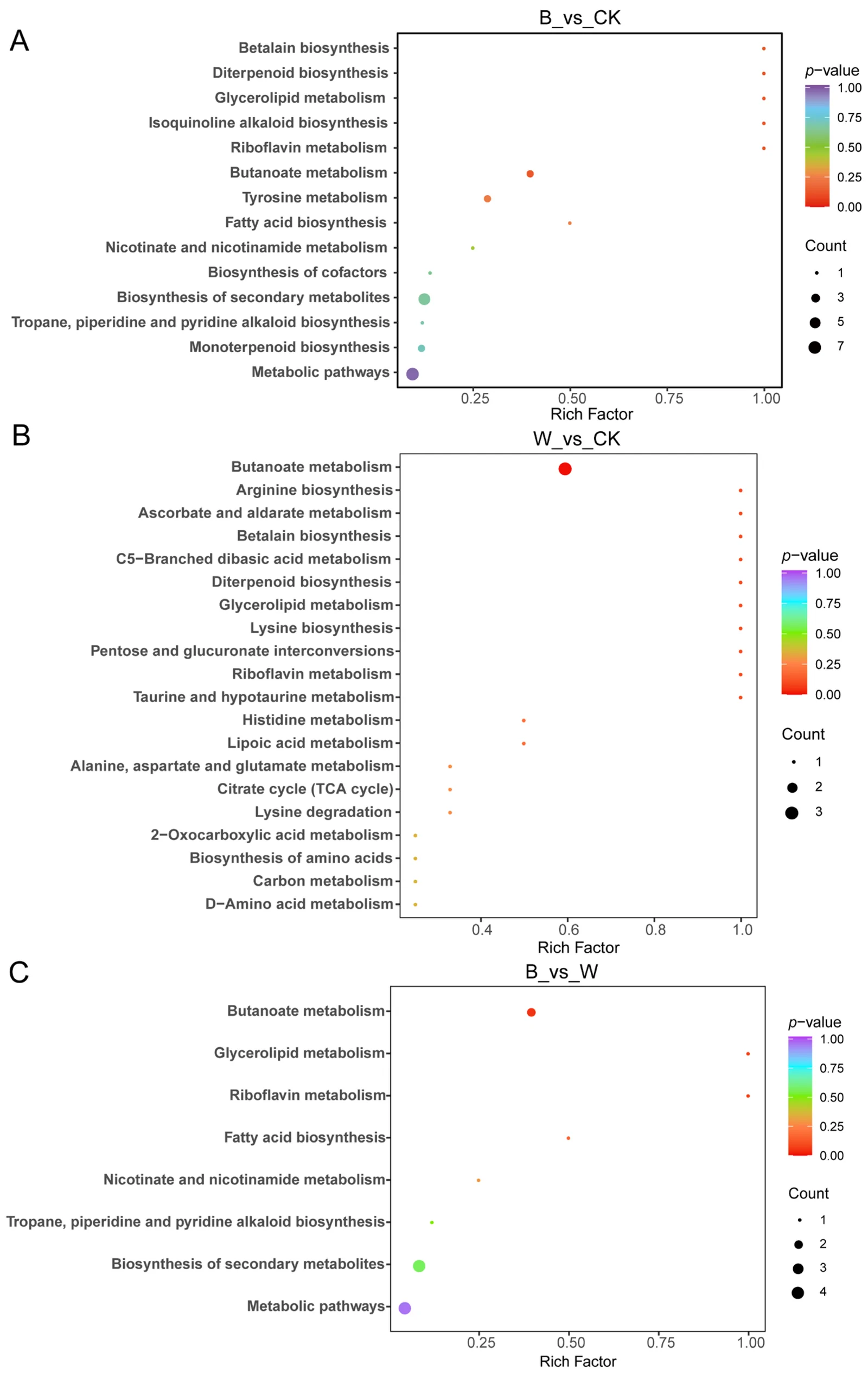
KEGG enrichment analysis of differential volatile metabolites (DVMs). (**A**–**C**) KEGG enrichment analysis of volatile metabolites for the B_vs_CK, W_vs_CK, and B_vs_W comparisons, respectively.

To investigate how different bagging treatments were associated with changes in volatile metabolism, KEGG pathway enrichment analysis was conducted for the differentially accumulated metabolites identified in the B vs. CK, W vs. CK, and B vs. W comparisons (Figure 5). KEGG enrichment analysis showed that the different bagging treatments markedly reshaped the metabolic networks associated with differential volatile metabolites in the peel of ‘Siam Red Pomelo’. Compared with the CK, the differential metabolites under the B treatment were mainly enriched in butanoate metabolism, fatty acid biosynthesis, glycerolipid metabolism, diterpenoid biosynthesis, monoterpenoid biosynthesis, tyrosine metabolism, and nicotinate and nicotinamide metabolism. Among these pathways, butanoate metabolism exhibited a relatively high enrichment level and contained a comparatively large number of differential metabolites, suggesting that the B treatment was associated with changes in pathways related to lipid, terpenoid, and short-chain fatty acid metabolism. In the comparison between the W treatment and CK, the differential metabolites were associated with a broader range of pathways, including butanoate metabolism; glycerolipid metabolism; diterpenoid biosynthesis; riboflavin metabolism; arginine, lysine, and histidine metabolism or biosynthesis; alanine, aspartate, and glutamate metabolism; the citrate cycle (TCA cycle); carbon metabolism; and ascorbate and aldarate metabolism. These results indicate that the W treatment not only affected the formation of lipid-derived volatiles but may also have altered amino acid metabolism, energy metabolism, and redox processes, thereby further regulating the accumulation of aroma precursors and related secondary metabolites. The differential metabolites between B and W were mainly enriched in butanoate metabolism, glycerolipid metabolism, fatty acid biosynthesis, riboflavin metabolism, nicotinate and nicotinamide metabolism, and the biosynthesis of secondary metabolites, with butanoate metabolism containing the largest number of differential metabolites. This finding indicates that the two bagging treatments generated distinct metabolic responses, which may result from differences in the physical properties and microenvironments created by the two bag types. Overall, bagging altered peel volatile composition mainly by affecting lipid, terpenoid, amino acid, and cofactor-related metabolic processes. The effects of the B treatment were relatively concentrated in lipid- and secondary metabolism-related pathways, whereas the W treatment induced broader responses involving primary and energy metabolism.

### 3.5. rOAV Distribution and Database-Derived Odor-Descriptor Analysis of Differential Volatile Metabolites

**Figure 6:**
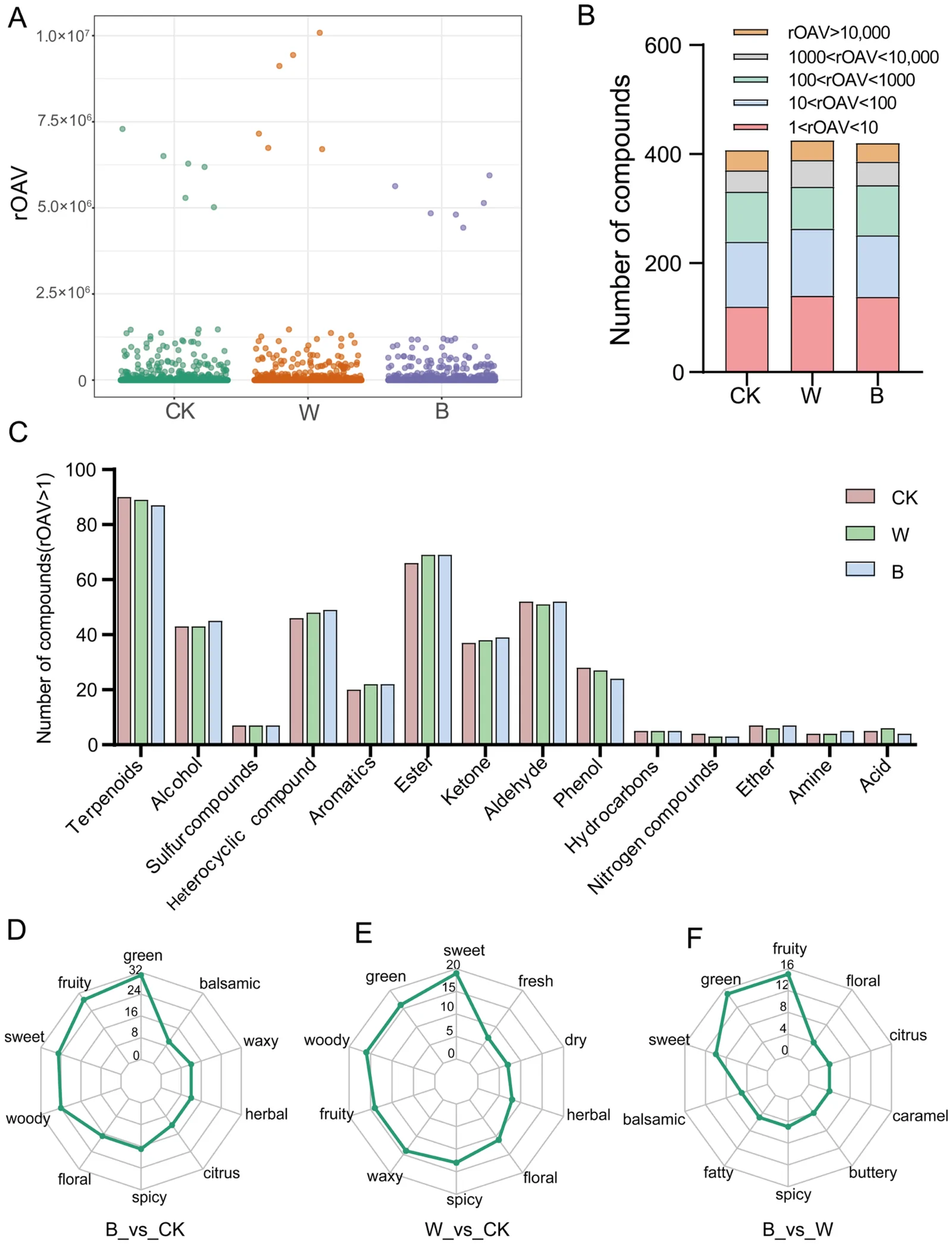
Effects of different bagging treatments on rOAV distributions and database-derived odor-descriptor associations of volatile compounds in pomelo fruit. (**A**) Distribution of relative odor activity values (rOAVs) of volatile compounds under the three treatments. Each point represents an individual volatile compound. (**B**) Numbers of volatile compounds within five rOAV ranges: 1–10, 10–100, 100–1000, 1000–10,000, and >10,000. (**C**) Distribution of volatile compounds with rOAV ≥ 1 across different chemical classes. (**D**–**F**) Radar plots showing the frequencies of database-derived odor descriptors associated with differential VOCs in the B vs. CK, W vs. CK, and B vs. W comparisons, respectively.

Different fruit-bagging treatments had only a limited effect on the overall chemical-class composition of pomelo peel VOCs but altered the rOAV distributions of individual compounds and their database-derived odor-descriptor associations. As shown in Figure 6A, most VOCs detected in the three treatments were distributed within the lower rOAV ranges, whereas only a small subset exhibited high rOAVs; these compounds were therefore prioritized for subsequent sensory-oriented validation rather than regarded as confirmed aroma contributors. The maximum rOAV in the W treatment exceeded 1.0 × 10^7^, which was higher than those in the CK treatment (approximately 7.2 × 10^6^) and the B treatment (approximately 5.9 × 10^6^). Although several high-rOAV compounds were also detected in the B treatment, its maximum rOAV was lower. These between-treatment differences describe the chemical screening profiles and should not be interpreted as direct sensory differences.

To prioritize VOCs with potential odor relevance under the different bagging treatments, rOAVs were calculated for the detected VOCs. VOCs with rOAV ≥ 1 were retained as candidate aroma-relevant VOCs for subsequent analysis. This threshold was used strictly as a chemical prioritization criterion and does not constitute direct sensory confirmation [32]. A total of 407, 425, and 420 VOCs with rOAV ≥ 1 were identified in the non-bagged control, W treatment, and B treatment, respectively, and were therefore retained as rOAV-prioritized candidates for subsequent analysis (Figure 6B and Table S2). In all treatments, compounds with 1 ≤ rOAV < 10 and 10 ≤ rOAV < 100 predominated, whereas relatively few compounds had rOAVs greater than 10,000. The total number of rOAV-prioritized VOCs was slightly higher in the W treatment, whereas that in the B treatment was comparable to that in the CK treatment. These results indicate treatment-dependent differences in the rOAV profiles of individual VOCs. Further sensory evaluation is required to determine whether these chemical differences translate into perceptible changes in fruit aroma.

Classification of the candidate aroma-active compounds with rOAVs ≥ 1 showed that terpenoids were the most abundant chemical class in all three treatments, comprising approximately 87–90 compounds, followed by esters, aldehydes, heterocyclic compounds, alcohols, and ketones (Figure 6C). This result indicates that terpenoids and esters were prominently represented among the candidate aroma-related VOCs selected by rOAV screening. Compared with the CK treatment, the numbers of terpenoids decreased slightly under the W and B treatments, with a relatively greater reduction observed in the B treatment. In contrast, the numbers of esters, heterocyclic compounds, aromatic compounds, and ketones increased slightly. Overall, the changes in the numbers of compounds within each chemical class were relatively small, indicating that bagging did not alter the fundamental compositional pattern of pomelo volatiles, which was dominated by terpenoids, esters, and aldehydes.

The distributions of database-derived odor descriptors associated with the differential VOCs are shown in Figure 6D–F. Differences between the B and CK treatments were mainly associated with green, fruity, sweet, and woody notes, with relatively large numbers of differential compounds related to green and fruity aromas. Differences between the W and CK treatments were primarily associated with sweet, green, woody, fruity, and waxy notes. However, the overall numbers of differential compounds corresponding to these aroma attributes were lower than those observed between the B and CK treatments. Differences between the B and W treatments were mainly concentrated in fruity, green, and sweet notes. These results demonstrate treatment-dependent differences in the distribution of VOCs associated with database-derived odor descriptors. Further sensory evaluation would be necessary to determine whether these differences are reflected in perceptible changes in the corresponding aroma characteristics.

In summary, bagging treatments had limited effects on the total number and major chemical classes of volatile compounds in pomelo fruit but altered the rOAV distribution of candidate aroma-related VOCs and the pattern of database-derived odor-descriptor associations. The W treatment exhibited a higher maximum rOAV for an individual compound and a slightly greater number of volatile compounds, while its overall database-derived odor-descriptor association pattern remained relatively similar to that of the CK treatment. In contrast, the B treatment was characterized by a lower maximum rOAV, a slight reduction in the number of terpenoids, and increased differences in compounds associated with green and fruity notes. These results provide a chemical basis for selecting treatment-responsive VOCs for subsequent sensory-oriented validation; they do not demonstrate treatment-induced perceptual aroma differences.

### 3.6. Database-Derived Odor-Descriptor Associations of Differential Volatile Metabolites Under Different Bagging Treatments

Differential volatile compounds were ranked according to their importance, and the top-ranked compounds were selected to construct Sankey diagrams based on their database-derived odor descriptors. Orange and purple links indicate metabolites that were upregulated and downregulated relative to the CK treatment, respectively. The Sankey diagrams therefore illustrate the annotation relationships between differential VOCs and their reported odor descriptors.

**Figure 7:**
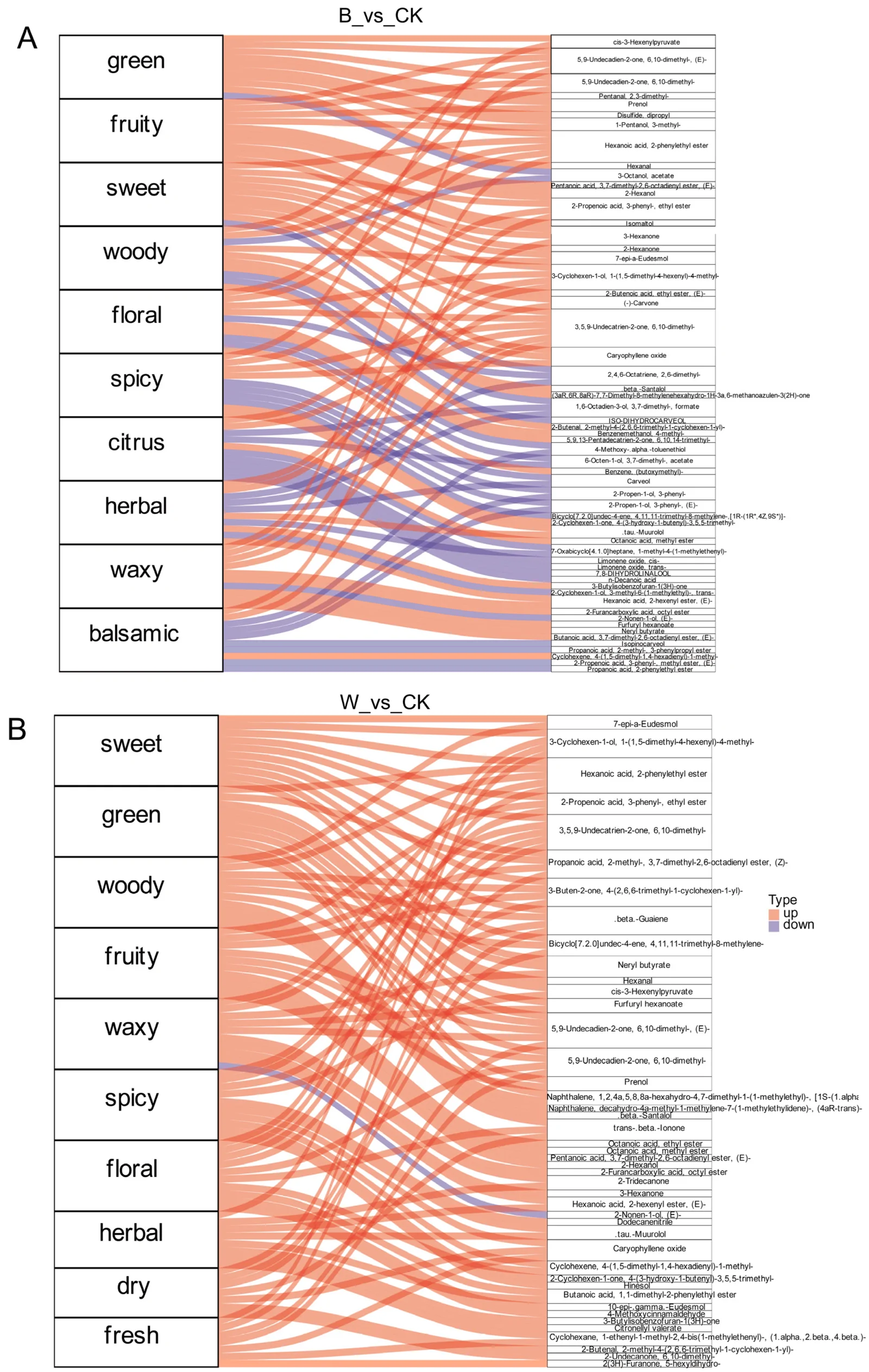
Flavor omics analysis of differential volatile metabolites. (**A**,**B**) Sankey diagrams illustrating the relationships between database-derived odor descriptors and differential volatile metabolites in the B vs. CK and W vs. CK comparisons, respectively.

In the comparison between the B and CK treatments, differential volatile compounds were mainly associated with green, fruity, sweet, woody, floral, spicy, citrus, herbal, waxy, and balsamic aroma attributes, with the strongest associations observed for green, fruity, and sweet aromas (Figure 7A). Compounds annotated with green, fruity, sweet, woody, floral, and spicy descriptors were predominantly upregulated, whereas those associated with citrus, herbal, waxy, and balsamic descriptors exhibited a relatively higher proportion of downregulation, indicating that the B treatment was associated with a distinct distribution of volatile compounds linked to these database-derived descriptors. The highest-ranked differential volatile compounds under the B treatment included 7-epi-α-eudesmol, ethyl cinnamate, isobutyl hexanoate, caryophyllene oxide, guaiacol, as well as several unsaturated ketones, terpenols, and esters (Figure 7A). Among these compounds, esters were mainly annotated with fruity and sweet descriptors, whereas terpenes and terpenols were associated with fruity, woody, floral, and herbal descriptors. Phenolic compounds were mainly associated with woody and spicy descriptors. The extensive overlap between individual volatile compounds and multiple odor descriptors indicates that the observed annotation pattern under the B treatment reflected diverse classes of differential VOCs. Further sensory analysis is needed to determine whether these compositional changes correspond to perceptible aroma differences.

Compared with the CK treatment, the differential volatile compounds identified under the W treatment were mainly associated with sweet, green, woody, fruity, and waxy odor descriptors, followed by spicy, floral, herbal, dry, and fresh descriptors, with the sweet descriptor showing the strongest association (Figure 7B). Most of the top-ranked differential volatile compounds were upregulated, whereas only a few waxy- and spicy-associated compounds were downregulated, indicating that the W treatment was characterized predominantly by increased accumulation of these descriptor-associated differential VOCs. Representative differential volatile compounds under the W treatment included 7-epi-α-eudesmol, phenethyl hexanoate, ethyl cinnamate, furfuryl hexanoate, hexyl butyrate, linalool, cis-3-hexenyl acetate, as well as several ionones, unsaturated ketones, and terpenes (Figure 7B). Esters were mainly annotated with fruity and sweet descriptors; linalool was associated with floral, sweet, and fresh descriptors; and cis-3-hexenyl acetate was associated with green, fruity, and fresh descriptors. The upregulation of these compounds was consistent with the greater representation of VOCs annotated with sweet, green, fruity, and floral descriptors.

Overall, the B treatment was characterized by both up- and downregulation of differential volatile compounds, resulting in a more dispersed modulation of aroma attributes. In contrast, the W treatment was dominated by the upregulation of differential volatile compounds, particularly those associated with sweet, green, fruity, and floral aromas. These results demonstrate distinct treatment-dependent distributions of differential VOCs and their database-derived odor-descriptor associations and provide candidate compounds for subsequent sensory validation.

## 4. Discussion

The composition and abundance of volatile metabolites constitute the chemical basis of fruit aroma quality. Citrus fruits are rich in volatile terpenes and also produce other classes of volatile compounds, including aromatics, alcohols, aldehydes, esters, and ketones. Among these compounds, terpenes represent the most abundant class of volatiles in citrus fruits. Metabolite identification in the present study showed that terpenes were the predominant class of volatile metabolites in the peel of ‘Siam Red Pomelo’, comprising 347 compounds and accounting for 21.71% of all identified metabolites. Further analysis based on the relative odor activity value (rOAV) revealed that terpenes also predominated among the VOCs in the highest rOAV range (rOAV ≥ 10,000). Compounds such as limonene, germacrene D, and (E)-β-damascenone consistently exhibited high rOAVs across the different treatments. These findings indicate that, although bagging altered the relative abundances of individual volatile metabolites, it did not change the overall terpene-dominated chemical profile or the prominence of terpenes among high-rOAV candidates in ‘Siam Red Pomelo’ peel. This result is consistent with previous reports showing that terpenes/terpenoids are predominant volatile constituents in citrus fruits, including yuzu (*Citrus junos*) from China [33], 108 accessions representing seven *Citrus* species/groups from China [7], ten major *Citrus* species worldwide [34], and Newhall navel orange (*Citrus sinensis* Osbeck var. brasiliensis Tanaka) from China [25]. However, previous studies also showed that the relative abundance of individual volatile compounds can vary considerably among citrus genotypes, tissues, and growing environments [7,25,33]. In the present study, bagging similarly altered the accumulation of specific volatile metabolites but did not change the overall terpene-dominated profile, suggesting that bagging mainly modulates the quantitative balance of volatile compounds rather than fundamentally reshaping the volatile composition of ‘Siam Red Pomelo’ peel. Moreover, the consistently high rOAVs of limonene, germacrene D, and (E)-β-damascenone show that rOAV-based prioritization depends on both semi-quantitative abundance and odor-threshold values, consistent with previous observations that low-abundance volatiles can nevertheless have low odor thresholds and potential sensory relevance [34].

Odor relevance cannot be established from volatile abundance alone because potential sensory impact depends on both concentration and odor threshold [35]. In the present study, rOAV was therefore used as a chemical screening metric to prioritize VOCs with potential odor relevance rather than as evidence that individual compounds directly determine fruit aroma characteristics. Consistent with previous studies showing that terpenes generally predominate in the volatile profiles of citrus fruits [36,37,38], the present study also showed that terpenes were prominently represented among the high-rOAV candidate VOCs of ‘Siam Red Pomelo’ peel. However, confirmation of the perceptual importance of individual high-rOAV compounds requires further evaluation using gas chromatography–olfactometry (GC–O), aroma recombination/omission experiments, and sensory assessment.

However, the high-rOAV candidate VOCs prioritized here differed from those reported for several other citrus species and cultivars. For example, previous studies reported relatively high accumulation of nootkatone in pomelos, whereas valencene, caryophyllene oxide, α-phellandrene, and trans-limonene oxide were abundant in sweet oranges, and several compounds, including citronellal, β-bisabolene, β-pinene, and α-terpineol, were relatively enriched in lemons [38]. In addition, limonene, α-pinene, β-pinene, 2-carene, 3-carene, γ-terpinene, and β-myrcene have been identified as major odor compounds in CFP [37], while β-myrcene, valencene, and trans-β-ocimene were proposed as characteristic aroma compounds of ‘Zongcheng’, and octanal, D-limonene, γ-terpinene, and methyl 2-(methylamino)benzoate as representative aroma compounds of ‘Lunwan’ [36]. In comparison, limonene, germacrene D, (E)-β-damascenone, linalool, β-ocimene, β-caryophyllene, and caryophyllene oxide exhibited high rOAVs in ‘Siam Red Pomelo’ peel in the present study. Thus, although our results partially overlap with previous reports, particularly with respect to limonene, β-ocimene, and caryophyllene-related compounds, the high rOAVs of germacrene D and (E)-β-damascenone contribute to a distinct high-rOAV VOC screening profile in ‘Siam Red Pomelo’. These differences further support the view that, despite the general predominance of terpenes in citrus volatiles, high-rOAV candidate VOC profiles are species- and cultivar-dependent. It should be emphasized that the present rOAV analysis was based on single-internal-standard semi-quantitative VOC estimates rather than compound-specific absolute quantification. Therefore, the calculated values are most appropriately interpreted as a comparative screening tool for prioritizing potentially odor-relevant VOCs. Confirmation of their perceptual importance will require GC–olfactometry, aroma recombination/omission experiments, and sensory evaluation. Despite this limitation, these compounds provide a valuable set of candidates for future sensory investigation of the aroma characteristics of ‘Siam Red Pomelo’ peel and for evaluating its potential as a raw material for essential oil extraction and processing.

The effect of bagging on volatile metabolism was characterized more by redistribution among individual metabolites and metabolic classes than by a uniform increase or decrease in volatile production. Although the total relative concentrations of VOCs were reduced by both bagging treatments, many individual metabolites showed increased accumulation. This apparent discrepancy suggests that bagging may reduce the abundance of a limited number of initially dominant compounds while promoting the relative accumulation of a broader set of low- or intermediate-abundance metabolites. Similar patterns have been observed in other fruits. In table grapes, bagging altered aroma characteristics by changing the proportions of aldehydes, alcohols, and esters without substantially altering total volatile content [15], while different colored paper bags produced distinct aroma profiles in ‘Kyoho’ grapes primarily through changes in the relative proportions of volatile classes [20]. Together, these findings indicate that bagging may reshape volatile composition mainly by redistributing metabolic flux among different volatile biosynthetic branches rather than by uniformly stimulating or suppressing volatile formation.

The two bagging treatments also produced distinct changes in the rOAV-prioritized candidate VOCs, indicating that bag properties influence not only the magnitude but also the direction of volatile metabolic responses. The B treatment relatively favored several sesquiterpenes and oxygenated sesquiterpenes, including β-caryophyllene, caryophyllene oxide, 7-epi-α-eudesmol, and guaiol, while reducing the contribution of some monoterpenes associated with green notes. These changes resulted in a greater representation of VOCs associated with woody descriptors and a lower representation of some green-associated VOCs. Because β-caryophyllene, caryophyllene oxide, 7-epi-α-eudesmol, and guaiol have been reported to possess woody or green odor characteristics, these compounds provide candidates for evaluating potential treatment-related sensory differences in future studies. Terpene biosynthesis in plants depends mainly on the methylerythritol phosphate (MEP) pathway in plastids and the mevalonate (MVA) pathway in the cytosol, which preferentially supply precursors for monoterpenes and carotenoids and for sesquiterpenes, respectively, although precursor exchange occurs between the two compartments [39,40]. Light availability and light quality can regulate genes encoding 1-deoxy-D-xylulose-5-phosphate synthase (DXS), 1-deoxy-D-xylulose-5-phosphate re-ductoisomerase (DXR), geranyl diphosphate synthase (GPPS) and terpene synthases, thereby influencing monoterpene formation and emission [41,42]. Therefore, the lower relative representation of monoterpenes and the higher relative representation of sesquiterpenes and their oxygenated derivatives under the B treatment may be associated with altered precursor allocation and terpene biosynthesis under the microenvironment created by the yellow–black double-layer paper bag treatment.

By contrast, the W treatment showed a volatile profile characterized by relatively greater contributions of linalool, β-ocimene, linalyl butyrate, and linalyl hexanoate, consistent with previous observations that the W treatment was associated with increased contributions of several monoterpene-related compounds, although the specific contribution of light conditions cannot be separated from other bag-related environmental factors. Linalool and β-ocimene are generally associated with floral characteristics, whereas linalool esters may contribute to sweet and fruity aroma attributes. In addition, the increased contribution of trans-β-ionone suggests that carotenoid-derived aroma formation may also have been affected. Unlike monoterpenes, trans-β-ionone is a C_13_ norisoprenoid generated through carotenoid cleavage by carotenoid cleavage dioxygenases (CCDs), and its accumulation is therefore influenced by both carotenoid availability and CCD activity [43,44]. The coordinated increases in volatile compounds associated with sweet, floral, and fruity odor descriptors under the W treatment may consequently reflect combined changes in monoterpene biosynthesis, carotenoid cleavage, and esterification rather than regulation of a single metabolic pathway.

The substantial overlap in differential metabolites between the two bagging treatments suggests that part of the volatile response represents a general effect of bagging, whereas treatment-specific metabolites indicate additional regulation associated with differences in bag properties. Similar common and treatment-dependent responses have been reported in bagged grapefruit and ‘Majiayou’ pomelo [45,46], while studies on grape, peach, and pear have shown that bag color, layer number, and light transmittance can differentially affect volatile, aroma-related, lipid, and wax metabolism [20,44,47,48]. Together with previous evidence that volatile responses to shading and bagging depend strongly on light intensity, treatment duration, and plant material [49], our results suggest that the W treatment and the B treatment created distinct fruit-surface microenvironments. These conditions may differentially regulate terpene precursor supply, terpene synthase activity, carotenoid cleavage, oxidation, and esterification, thereby reshaping the relative proportions of monoterpenes, sesquiterpenes, oxygenated terpenes, carotenoid-derived volatiles, and esterified terpene derivatives. Future studies integrating metabolite profiling with the expression and enzymatic activities of DXS, DXR, 4-hydroxy-3-methylbut-2-enyl diphosphate reductase (HDR), 3-hydroxy-3-methylglutaryl-CoA reductase (HMGR), GPPS, farnesyl diphosphate synthase (FPPS), terpene synthases, CCDs, and relevant acyltransferases will be required to clarify the molecular mechanisms underlying these bagging-induced changes.

## 5. Conclusions

This study systematically characterized the peel volatilome and screened candidate aroma-relevant VOCs of ‘Siam Red Pomelo’ under different fruit-bagging treatments. HS-SPME–GC–MS profiling showed that terpenoids and esters were major components of the volatile profile, while rOAV analysis was used to prioritize VOCs with potential aroma relevance. Different bagging treatments were associated with distinct volatile metabolic patterns: the yellow–black double-layer paper bag treatment showed greater relative representation of several sesquiterpenes and oxygenated derivatives, whereas the white nonwoven fabric bag treatment showed greater relative representation of several VOCs associated with database-derived floral and fruity odor descriptors. KEGG analysis further indicated that bagging-treatment-associated differences involved terpenoid, lipid, and butanoate metabolism. These findings demonstrate that different bag types are associated with distinct changes in peel volatile metabolism and composition in ‘Siam Red Pomelo’. The rOAV results and database-derived odor-descriptor associations provide a chemical basis for hypotheses about possible treatment-related aroma differences, but they do not constitute direct sensory evidence. Because the rOAVs were derived from single-internal-standard semi-quantitative VOC estimates and no GC–olfactometry, sensory evaluation, or aroma recombination/omission experiments were conducted, whether these chemically inferred differences are perceptible remains unresolved and requires sensory validation using those approaches.

## Data Availability

The original contributions presented in this study are included in the article/Supplementary Material. Further inquiries can be directed to the corresponding authors.

## Supplementary Materials

The following supporting information can be downloaded at https://www.mdpi.com/article/10.3390/foods15203608/s1, Figure S1. Top 20 differentially abundant volatile metabolites. B: yellow–black double-layer paper bag providing strong shading; W: white nonwoven fabric bag providing moderate shading; CK: non-bagged control. Table S1 Classification of differential volatile metabolites among groups. Table S2 Classification of volatile metabolites with rOAV > 1 under different treatments. Table S3 _B_vs_CK_flavor_count. Table S4_W_vs_CK_flavor_count. Table S5_B_vs_W_flavor_count.

## Abbreviations

The following abbreviations are used in this manuscript:

rOAV	Relative Odor Activity Value
HS-SPME-GC-MS	HeadSpace Solid Phase Microextraction–Gas Chromatography Mass Spectrometry
B	yellow–black double-layer paper bag treatment
W	white nonwoven fabric bag treatment
CK	non-bagged control

## References

[1] Lu X., Zhao C., Shi H., Liao Y., Xu F., Du H., Xiao H., Zheng J. (2023). Nutrients and Bioactives in Citrus Fruits: Different Citrus Varieties, Fruit Parts, and Growth Stages. Crit. Rev. Food Sci. Nutr..

[2] Wu G.A., Terol J., Ibanez V., López-García A., Pérez-Román E., Borredá C., Domingo C., Tadeo F.R., Carbonell-Caballero J., Alonso R. (2018). Genomics of the Origin and Evolution of Citrus. Nature.

[3] Promkaew P., Pongprasert N., Wongs-Aree C., Kaewsuksaeng S., Opio P., Kondo S., Srilaong V. (2020). Carotenoids Accumulation and Carotenoids Biosynthesis Gene Expression during Fruit Development in Pulp of Tubtim-Siam Pummelo Fruit. Sci. Hortic.-Amsterdam..

[4] Schuman M.C. (2023). Where, When, and Why Do Plant Volatiles Mediate Ecological Signaling? The Answer Is Blowing in the Wind. Annu. Rev. Plant Biol..

[5] Mostafa S., Wang Y., Zeng W., Jin B. (2022). Floral Scents and Fruit Aromas: Functions, Compositions, Biosynthesis, and Regulation. Front. Plant Sci..

[6] Zhang Y., Luo R., Yang R., Ma X., Wang M. (2026). Citrus Essential Oils: A Review of Chemical Composition, Bioactivity, Safe Use, and Potential Applications. Chem. Biodivers..

[7] Zhang H., Xie Y., Liu C., Chen S., Hu S., Xie Z., Deng X., Xu J. (2017). Comprehensive Comparative Analysis of Volatile Compounds in Citrus Fruits of Different Species. Food Chem..

[8] Cui Q., Jiang L.-J., Wen L.-L., Tian X.-L., Yuan Q., Liu J.-Z. (2024). Metabolomic Profiles and Differential Metabolites of Volatile Components in Citrus Aurantium Changshan-Huyou Pericarp during Different Growth and Development Stages. Food Chem. X.

[9] Duan Y., Liu Z., Zhang Q., Hu J., Zheng S., Mao X., Wang Y. (2025). Dynamic Changes in Volatile and Non-Volatile Components of Jinggang Honey Pomelo (*Citrus maxima* (L.) Osbeck) during Growth Revealed through an Untargeted Metabolomics Approach. Food Chem..

[10] He X., Gong C., Zhang X., Li L., He Y., Zhao G., Chen C., Wang Q., Kong X., Hong G. (2026). Integrated Metabolomics and Transcriptomics Reveal the Molecular Basis of Cultivar-Specific Floral Scent Biosynthesis in Fingered Citron (*Citrus medica*). Ind. Crops Prod..

[11] Cao K., Yao C., Xiao J., Huang R., Qin Z., Shi Y., Chen J., Li L. (2026). Elucidation of Dynamic Evolution Patterns of Non-Volatile and Volatile Compounds in Peel of Citrus Reticulata Cv. Chachiensis with Different Tree Ages: A Metabolomics-Based Analysis. Food Res. Int..

[12] Hou J., Liang L., Wang Y. (2020). Volatile Composition Changes in Navel Orange at Different Growth Stages by HS-SPME–GC–MS. Food Res. Int..

[13] Xie L., Dong Y., Qin Q., Jiang C., Shang L., Qiao K., Wang B., Zeng J., Luo Q., Zhong L. (2026). Integrated Flavoromics and Metabolomics Reveal Aroma and Bioactive Characteristics of Six Mainstream Citri Reticulatae Pericarpium Cultivars. Foods.

[14] Ali M.M., Anwar R., Yousef A.F., Li B., Luvisi A., De Bellis L., Aprile A., Chen F. (2021). Influence of Bagging on the Development and Quality of Fruits. Plants.

[15] Andreu-Coll L., Noguera-Artiaga L., Sendra E., Hernández F. (2025). Impact of Preharvest Bagging on the Volatile Profile of Vinalopó Table Grapes. Agronomy.

[16] Li C., Li X., Liang G., Xiang S., Han G. (2025). Analysis of Phenols and Volatile Metabolites during Lemon Fruit Ripening after Bagging Treatment. Hortscience.

[17] Tang Y., Yao Y., Wu Y., Yang S. (2025). The Volatile Composition, Biosynthesis Pathways, Breeding Strategies, and Regulation Measures of Apple Aroma: A Review. Horticulturae.

[18] Wang Y., Yang C., Liu C., Xu M., Li S., Yang L., Wang Y. (2010). Effects of Bagging on Volatiles and Polyphenols in “Wanmi” Peaches during Endocarp Hardening and Final Fruit Rapid Growth Stages. J. Food Sci..

[19] He L., Xu X.-Q., Wang Y., Vanderweide J., Sun R.-Z., Cheng G., Chen W., Li S.-D., Li S.-P., Duan C.-Q. (2022). Differential Influence of Timing and Duration of Bunch Bagging on Volatile Organic Compounds in Cabernet Sauvignon Berries (*Vitis vinifera* L.). Aust. J. Grape Wine Res..

[20] Ji X., Wang B., Wang X., Shi X., Liu P., Liu F., Wang H. (2019). Effects of Different Color Paper Bags on Aroma Development of Kyoho Grape Berries. J. Integr. Agric..

[21] Feng C., Zhang X., Wang J., Wu C., Wang W., Yan G., Zhou Y., Zhang K., Duan X. (2025). Characterization of Volatile Profiles in Cherry Fruits: Integration of E-Nose and HS-SPME-GC–MS. Food Chem. X.

[22] Li X., Li G., Wang H., Liu C., Rong C., Song F., Chen C., Wu J. (2025). Characterization of Volatile Flavor Profiles in Three Peach Cultivars during Postharvest Storage at Various Temperatures Using HS-SPME-GC–MS. Food Chem. X.

[23] Xu L., Liu W., Pan Z., Pang F., Zhang Y., Liang J., Wang Q., Wang J., Zhao M., Qiao Y. (2025). Characterization and Comparative Analysis of Volatile Organic Compounds in Four Aromatic Wild Strawberry Species Using HS-SPME-GC-MS. Food Chem. X.

[24] Zhang Y., Yan Y., Shu H., Dan Q., Pan L., Tu K. (2025). Analysis of Aroma and Key Differential Volatile Compounds during Ripening Stages of Three Strawberry Cultivars Using HS-SPME-GC–MS, GC-IMS, and E-Nose. Food Chem..

[25] Hu Y., Lu W., Ma M., Wang J., Ma Y., Zheng Y. (2026). Geographical Variations of Volatile Metabolites in Newhall Navel Orange Based on HS-SPME-GC-MS and Meteorological Factors. Foods.

[26] Liu D., Shen Q., Lin K., Wang F., Bu Z., Peng J., Brennan C., Benjakul S., Xiao G., Ma L. (2024). The Aroma Profiles of Dried Gonggans: Characterization of Volatile Compounds in Oven-Dried and Freeze-Dried Gonggan. Food Res. Int..

[27] Du Y., Zhang H., Rothenberg D.O.N., Zhang K., Lin Y., Jin K., Li J., Zhou H., Su H., Ji R. (2024). Multiomics Correlation Analysis on the Mechanism of Flavor Substance Formation during the Processing of “huanong 53” Black Tea. LWT.

[28] Chen L., Song H., Xin J., Cheng W., Yang M., Sun H. (2025). Unraveling the Floral Aroma of Lotus (*Nelumbo nucifera*): Insights from Volatile Metabolomics and Transcriptomics. Ind. Crops Prod..

[29] Yuan H., Jiangfang Y., Liu Z., Su R., Li Q., Fang C., Huang S., Liu X., Fernie A.R., Luo J. (2024). WTV2.0: A High-Coverage Plant Volatilomics Method with a Comprehensive Selective Ion Monitoring Acquisition Mode. Mol. Plant.

[30] Yuan H., Cao G., Hou X., Huang M., Du P., Tan T., Zhang Y., Zhou H., Liu X., Liu L. (2022). Development of a Widely Targeted Volatilomics Method for Profiling Volatilomes in Plants. Mol. Plant.

[31] Huang D., Chen X., Tan R., Wang H., Jiao L., Tang H., Zong Q., Mao Y. (2024). A Comprehensive Metabolomics Analysis of Volatile and Non-Volatile Compounds in Matcha Processed from Different Tea Varieties. Food Chem. X.

[32] Yang Y., Ai L., Mu Z., Liu H., Yan X., Ni L., Zhang H., Xia Y. (2022). Flavor Compounds with High Odor Activity Values (OAV > 1) Dominate the Aroma of Aged Chinese Rice Wine (Huangjiu) by Molecular Association. Food Chem..

[33] Liu C., Xiao Y., Zhang J., Lu S., Zheng M., Wu Q., Cao Y., Xia Q. (2026). Comparison of Chemical Profiles and Identification of Key Bioactive Compound in Yuzu (*Citrus junos*) Pulp and Peel. Food Res. Int..

[34] González-Mas M.C., Rambla J.L., López-Gresa M.P., Amparo Blázquez M., Granell A. (2019). Volatile Compounds in Citrus Essential Oils: A Comprehensive Review. Front. Plant Sci..

[35] Van Ruth S.M. (2001). Methods for Gas Chromatography-Olfactometry: A Review. Biomol. Eng..

[36] Hu Z., Chen M., Zhu K., Liu Y., Wen H., Kong J., Chen M., Cao L., Ye J., Zhang H. (2024). Multiomics Integrated with Sensory Evaluations to Identify Characteristic Aromas and Key Genes in a Novel Brown Navel Orange (*Citrus sinensis*). Food Chem..

[37] Moon H.S., Hong S.J., Yu S.Y., Park H., Ban Y., Jang G.-J., Kim H.E., Lee S.-H., Shin E.-C. (2026). Elucidation of Flavor Codes in Citrus Fruit Peels Using Electronic Sensors, LC-MS/MS, and GC–MS Combined with GC-O. Food Chem. X.

[38] Zhang H., Wen H., Chen J., Peng Z., Shi M., Chen M., Yuan Z., Liu Y., Zhang H., Xu J. (2019). Volatile Compounds in Fruit Peels as Novel Biomarkers for the Identification of Four Citrus Species. Molecules.

[39] Dudareva N., Andersson S., Orlova I., Gatto N., Reichelt M., Rhodes D., Boland W., Gershenzon J. (2005). The Nonmevalonate Pathway Supports Both Monoterpene and Sesquiterpene Formation in Snapdragon Flowers. Proc. Natl. Acad. Sci. USA.

[40] Laule O., Fürholz A., Chang H.S., Zhu T., Wang X., Heifetz P.B., Gruissem W., Lange B.M. (2003). Crosstalk between Cytosolic and Plastidial Pathways of Isoprenoid Biosynthesis in Arabidopsis Thaliana. Proc. Natl. Acad. Sci. USA.

[41] Han J., Li T., Wang X., Zhang X., Bai X., Shao H., Wang S., Hu Z., Wu J., Leng P. (2022). AmMYB24 Regulates Floral Terpenoid Biosynthesis Induced by Blue Light in Snapdragon Flowers. Front. Plant Sci..

[42] Yu X., Qi X., Li S., Fang H., Bai Y., Li L., Liu D., Chen Z., Li W., Liang C. (2021). Transcriptome Analysis of Light-Regulated Monoterpenes Biosynthesis in Leaves of *Mentha canadensis* L. Plants.

[43] Liu H., Cao X., Azam M., Wang C., Liu C., Qiao Y., Zhang B. (2022). Metabolism of Carotenoids and β-Ionone Are Mediated by Carotenogenic Genes and PpCCD4 under Ultraviolet B Irradiation and during Fruit Ripening. Front. Plant Sci..

[44] Wang X.-Y., Wang H.-L., Zhang G.-J., Yan A.-L., Ren J.-C., Liu Z.-H., Xu H.-Y., Sun L. (2022). Effects of Fruit Bagging Treatment with Different Types of Bags on the Contents of Phenolics and Monoterpenes in Muscat-Flavored Table Grapes. Horticulturae.

[45] Jiang A., Zheng L., Wang D., Kang C., Wu J., Fang P., Cao J., Sun C. (2022). Effects of Fruit Bagging on the Physiochemical Changes of Grapefruit (*Citrus paradisi*). Food Qual. Saf..

[46] Jiang Q., Ye J., Zhu K., Wu F., Chai L., Xu Q., Deng X. (2022). Transcriptome and Co-Expression Network Analyses Provide Insights into Fruit Shading That Enhances Carotenoid Accumulation in Pomelo (*Citrus grandis*). Hortic. Plant J..

[47] Jia H.J., Araki A., Okamoto G. (2005). Influence of Fruit Bagging on Aroma Volatiles and Skin Coloration of “hakuho” Peach (*Prunus persica* Batsch). Postharvest Biol. Technol..

[48] Wu X., Lei Z., Yuan Y., Shi X., Chen Y., Qi K., Xie Z., Bai M., Yin H., Zhang S. (2024). Integrated Metabolomic and Transcriptomic Analysis Revealed the Role of PbrCYP94B in Wax Accumulation in Pear Fruit after Bagging Treatment. Int. J. Biol. Macromol..

[49] Bureau S.M., Baumes R.L., Razungles A.J. (2000). Effects of Vine or Bunch Shading on the Glycosylated Flavor Precursors in Grapes of *Vitis vinifera* L. Cv. Syrah. J. Agric. Food Chem..

