# Supplementary material for: Volatile Metabolomics Reveals the Effects of Different Bagging Types on Peel Aroma of ‘Siam Red Pomelo’
Source: Foods. 2026 Oct 10;15(20):3608. doi: 10.3390/foods15203608 (PMC13703433; doi:10.3390/foods15203608)
Supplement: Supplementary file 1 [file foods-15-03608-s001.zip › 1-Supplementary figure.pdf]

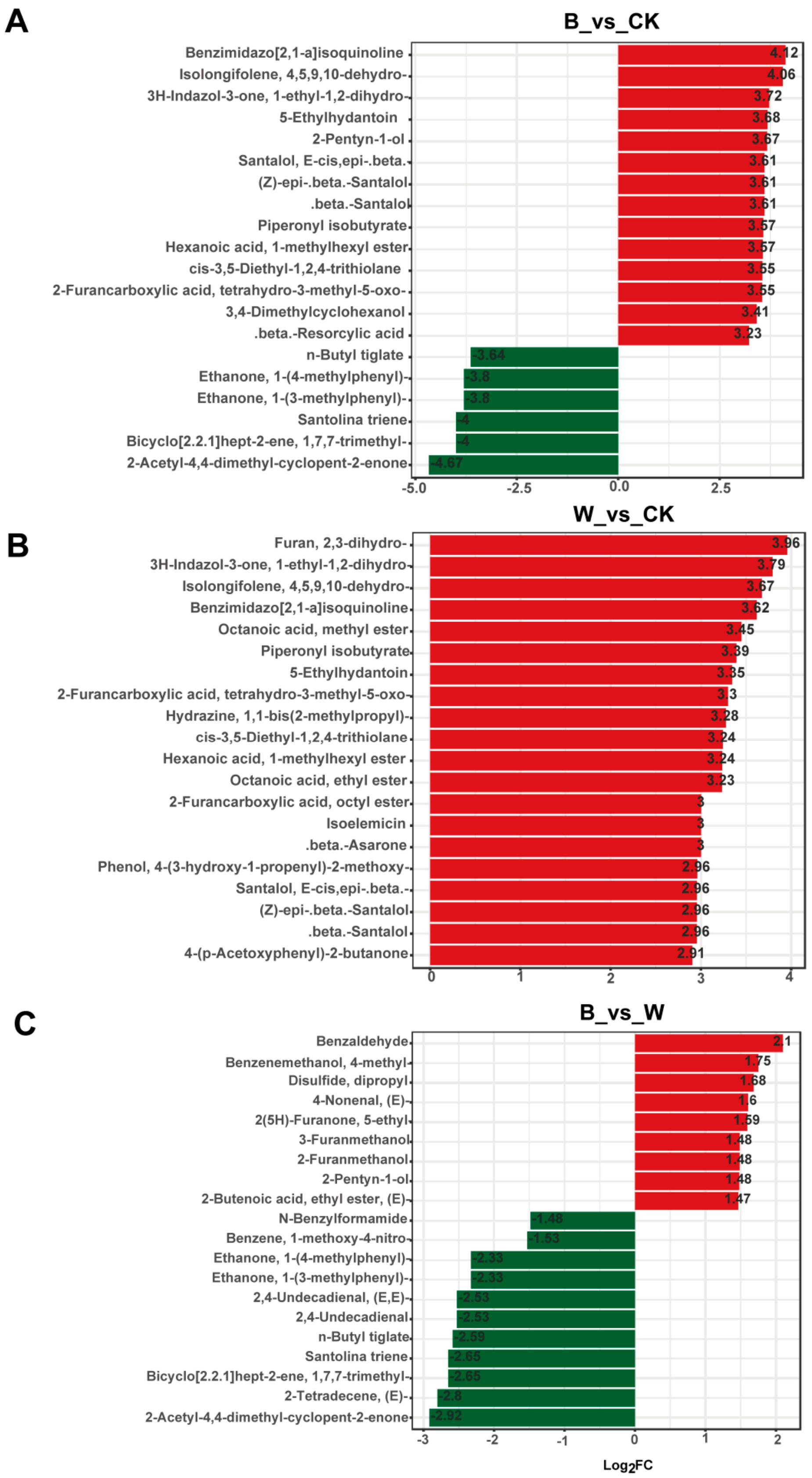

Figure S1. Top 20 differentially abundant volatile metabolites. B: yellow – black double-layer paper bag providing strong shading; W: white nonwoven fabric bag providing moderate shading; CK: non-bagged control.

Table S1 Classification of differential volatile metabolites among groups.

	B_vs_CK		W_vs_CK		B_vs_W	
	**up**	**down**	**up**	**down**	**up**	**down**
Terpenoids	33	26	36	1	0	11
Sulfur compounds	2	0	0	0	1	0
Phenol	2	3	3	0	0	0
Nitrogen compounds	2	0	3	0	0	1
Ketone	33	7	21	3	12	4
Hydrocarbons	12	4	7	1	4	4
Heterocyclic compound	16	8	13	1	4	4
Ether	4	2	3	0	0	1
Ester	25	17	24	3	4	13
Amine	4	5	3	0	1	2
Aldehyde	7	4	5	1	4	2
Alcohol	32	5	21	1	26	4
Acid	9	3	8	2	0	2

Table S2 Classification of volatile metabolites with rOAV > 1 under different treatments.

**Class I**	**CK**	W	B
Terpenoids	90	89	87
Alcohol	43	43	45
Sulfur compounds	7	7	7
Heterocyclic compound	46	48	49
Aromatics	20	22	22
Ester	66	69	69
Ketone	37	38	39
Aldehyde	52	51	52
Phenol	28	27	24
Hydrocarbons	5	5	5
Nitrogen compounds	4	3	3
Ether	7	6	7
Amine	4	4	5
Acid	5	6	4
